# Light People: Professor Manijeh Razeghi

**DOI:** 10.1038/s41377-023-01343-8

**Published:** 2024-07-15

**Authors:** Hui Wang, Cun Yu

**Affiliations:** Changchun Institute of Optics, Fine Mechanics and Physics, Chinese Academy of Sciences, Changchun, 130033 China

**Keywords:** Quantum dots, Terahertz optics

## Abstract

The sense of light is the first sensation the human body develops. The importance of light is self-evident. However, we all know that the light we can see and perceive covers only a small section of the spectrum. Today, for Light People, we feature a researcher who is committed to exploring different spectral bands of light ranging from deep ultraviolet to terahertz waves and working on quantum semiconductor technology, Prof. Manijeh Razeghi of the Northwestern University in the United States. Known for her quick thinking and witty remarks, Prof. Razeghi is passionate about life and always kind to others. As a scientist, she does not limit her research to a single focus, instead, she works on the entire process from material selection, device design, processing, and manufacturing, all the way to product application. She has a strong passion for education, a commitment unwavered by fame or fortune. For her students, she is both a reliable source of knowledge and a motherly figure with a caring heart. She firmly believes that all things in nature can give her energy and inspiration. In science, she is a true “pioneer” in research and a “miner” of scientific discoveries. She advises young scientists to enjoy and love what they do, and turn their research into their hobby. As a female scientist, she calls on all women to realize their true value and potential. Next, let’s hear from Professor Manijeh Razeghi, a true star who radiates energy and light.

## Abstract (web summary)

Prof. Manijeh Razeghi, a true star who radiates energy and light, calls on all women to realize their true value and potential.

## Graphical abstract

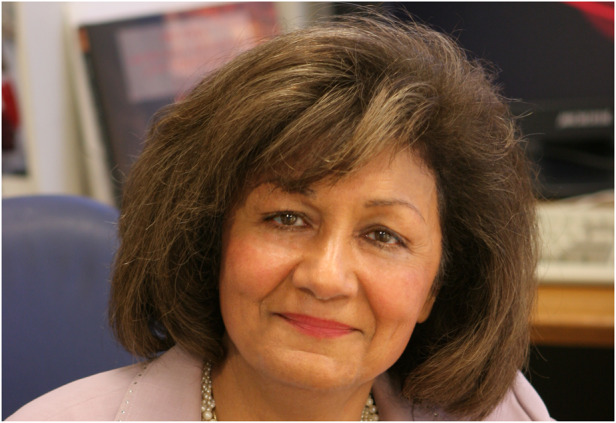


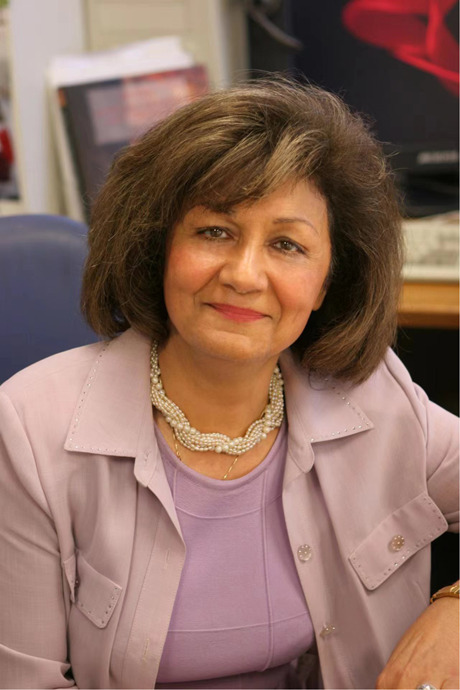


Prof. Manijeh Razeghi received the Doctorate d’état ES Sciences Physiques from the Université de Paris, France, in 1980.

She joined Northwestern University, Evanston, IL, as a Walter P. Murphy Professor and Director of the Center for Quantum Devices in the fall 1991, where she created the undergraduate and graduate program in solid-state engineering.

She has authored or co-authored more than 1,000 papers, more than 35 book chapters, and 20 books, including 4 textbooks: *Technology of Quantum Devices* (Springer Science Business Media, Inc., New York, NY, USA, 2010) and *Fundamentals of Solid-State Engineering,* *4*^*th*^ *Edition* (Springer Science Business Media, Inc., New York, NY, USA, 2018). *The MOCVD Challenge*, *2*^*nd*^ *Edition* (Taylor & Francis/CRC Press, 2010) represents the combined updated version of Volumes 1 and 2. And *the Mystery of Carbon* IOP 2019. She holds many U.S. patents and has given more than 1000 invited and plenary talks. Her current research interest is in nanoscale optoelectronic quantum devices, from deep UV to THz.

Prof. Razeghi is a Fellow of MRS, IOP, IEEE, APS, SPIE, OSA, Fellow and Life Member of Society of Women Engineers (SWE), and Fellow of the International Engineering Consortium (IEC). She received the IBM Europe Science and Technology Prize in 1987, the Achievement Award from the SWE in 1995, the R.F. Bun shah Award in 2004, IBM Faculty Award 2013, the Jan Czochralski Gold Medal in 2016, the 2018 Benjamin Franklin Medal in Electrical Engineering, LSA 10^th^ Anniversary Outstanding Contribution Award, and many best paper awards. She is an elected life-Fellow of SWE, IEEE, and MRS. She is honored as a member of the Academy of Europe 2021.

**1. Could you briefly introduce your current research direction and main focus please?**

Prof. Razeghi: Nature is based on Quantum, our body, our brain, all of human senses are based on Quantum. We try to learn more and more about and be inspired by nature. We try to mimic nature. Nature provides us the full assortment of atoms in the periodic table, these atoms are the building blocks of all matters, and work hand in hand with their partner photon, a piece of light to communicate energy from one atom to another.

When nature binds atoms together, the combinations provide nature with a rich palette of colors to decorate the world around us and give rise to the functional complexity of nature. The wings of a butterfly, the feather of a peacock, the sheen of a pearl ... all of these are examples of photonic crystals: nanostructured arrangements of atoms that capture and recast the colors of the rainbow with iridescent beauty.

Different atoms emit light of different colors or energy when oscillating—from gamma rays, X-Rays, Ultraviolet (UV), visible and through invisible infrared (IR) rays down to THz. However, our eyes can see only a small part of the total electromagnetic spectrum: the colors of the rainbow.

Physicists have discovered that detecting and creating light of different colors require first a profound understanding of the structure of the atom through Quantum Mechanics, and a special type of materials called semiconductors, the heart of all modern electronics, and photonics. Thus, to see beyond the visible wavelengths, scientists have invented artificial eyes, such as infrared cameras.

However, there still exist frontiers within the invisible light spectrum that we still cannot detect. To both see and shine light in either of these wavelength bands requires a team of scientists to invent new models, materials, and devices. This is what my group at Center for Quantum Device (CQD) is dedicated to exploring these frontiers of the invisible light spectrum by developing quantum semiconductor technologies to both emitting and detecting light of different colors from deep UV to THz.

**Figure:**
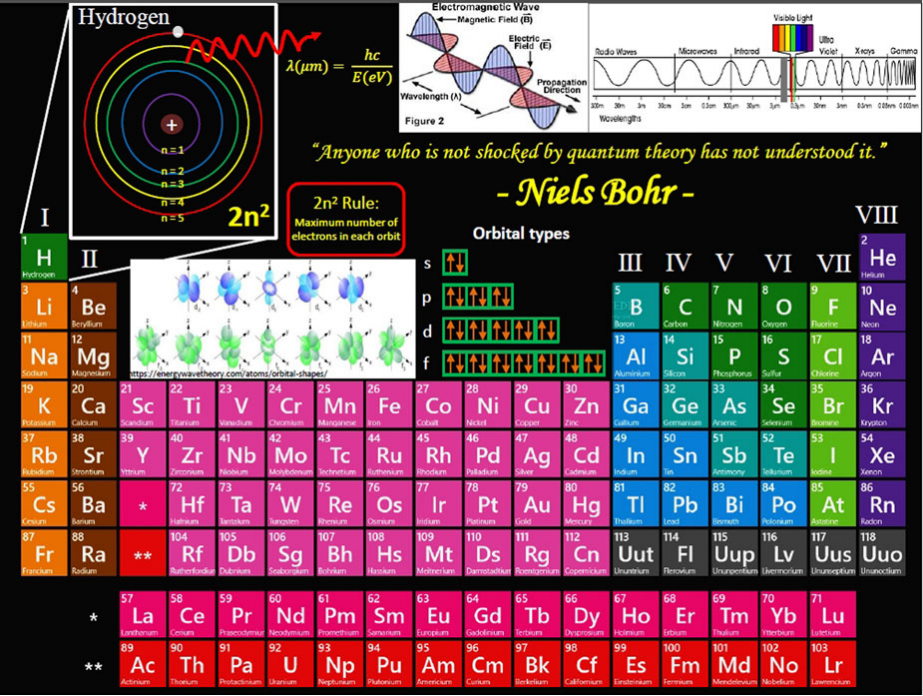
Nature provides us the full assortment of atoms in periodic table

**Figure:**
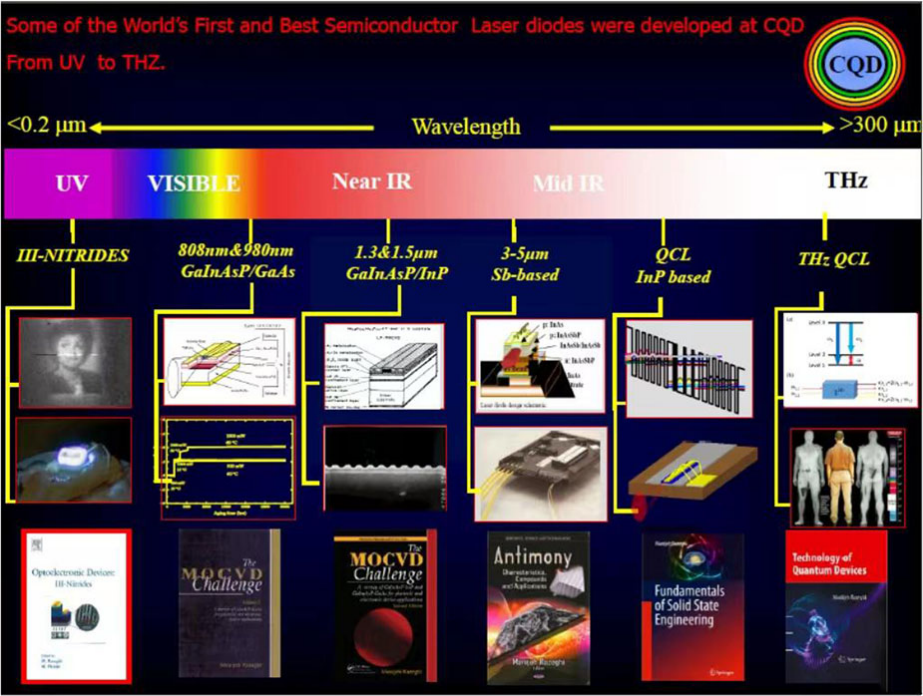
Some of the world’s first and best semiconductor laser diodes were developed by Prof. Razeghi at Thomson CSF in France (1981–1991), and CQD/Northwestern University

**2. Your research covers material selection, device design, processing and manufacturing, and product application. Which of these do you find most challenging? How did you and your team overcome the difficulties?**

Prof. Razeghi: First, let me start with deep UV. These are very short wavelengths with very high photon energies, making it one of the most challenging wavelength band to build quantum semiconductor detectors, lasers, and modulators.

To detect and emit light at the deep-UV range, which has many applications, I led the designing of a specialized epitaxial reactor that can grow, atom-by-atom, the semiconductor materials to build these UV-light-emitting diodes, detectors, and lasers.

One of the important features of these deep UV semiconductor quantum devices in my lab is the ability to disinfect surfaces just by shining UV light on them. The light in this band can deactivate the DNA of bacteria, viruses and other pathogens and thus destroy their ability to multiply and cause disease. For instance, it was found that a deep UV light source that we developed (for the first time in the world), kills >99% of the COVID-19 virus on a surface in just 1 s of shining! This UV light can also kill viruses in the air we breathe.

By pairing the UV light with UV detectors (also developed by us and first in the world), we can create a system that rapidly detects and identifies certain harmful biological agents such as Anthrax, Tyrosine, COVID-19, etc., in the air and on surfaces. This is an important application for health and safety and is still one of the most important research subjects in my group and around the world.

The second important research in my group is in the terahertz frequency band. These are very long wavelengths with low photon energies, also making it one of the most challenging wavelengths for which to build room-temperature quantum semiconductor detectors and laser diodes.

The unique epitaxial reactors that I led the design of can build the quantum semiconductor materials, required to create THz detector and lasers diodes for this subject. The pairing of exquisite quantum semiconductor materials with new innovative laser designs has led my group to demonstrate the first room temperature, compact THz laser source. These THz semiconductor laser diodes, detectors, and Quantum eyes could have far-reaching impacts in healthcare and global communications over the next decade and beyond.

Light at THz frequencies has many unique properties. It can pass through a variety of substances, including synthetics, textiles, paper, and cardboard. Unlike X-rays, THz waves do not have any ionizing effects due to their low energy and are generally considered biologically harmless. This makes THz waves much safer and healthier than X-rays when used for security checking and medical diagnosis.

The second future application is in the area of high-speed communications. Since THz frequencies are orders of magnitude higher than those used for the current wireless communication, there is the potential to apply THz technology for the next generation of high-speed wireless communications. The higher the frequency of the “light”, the faster the data is sent and information can be delivered.

Our goal is to create a compact, mass-producible, and room temperature THz Laser source with high output power in order to enable technology for the applications mentioned above, and so many others.

Epitaxy of high-quality material is the most challenging and the most important technology. Every high-power laser, high-performance detector relies on this well-refined epitaxy technology which is the foundation of all the above subjects.

**3. Four years ago, you called InAs/GaSb type II superlattices the third generation of infrared imaging materials. What are its advantages over other materials?**

Prof. Razeghi: The Sb-based bandgap-engineered Type II superlattices (T2SLs) have drawn a lot of attention since they were introduced in 1970 by Leo Ezaki et al., for infrared detection as a system of multi‐interacting quantum wells. T2SL material system has experienced incredible improvements in design, gap and atomic engineering, high-quality materials, device structure designs and device fabrication process, which elevated the performances of T2SL‐based photodetectors to a comparable or superior level to the state-of-the-art material systems for infrared detection such as Mercury Cadmium Telluride (MCT). As a pioneer in the field, CQD has been involved in growth, design, characterization, and introduction of Sb-based Bulk and T2SL material system for infrared photodetection and imaging from deep UV to THz.

Currently, many infrared focal planar arrays are based on MCT II–VI (with ionic bonding) material system. Although it has a higher detection efficiency, its poor material uniformity, low device yield, and environmental hazardousness are limiting its large-scale application.

Based on bandgap engineering, InAs/GaSb type II superlattice possesses an adjustable bandgap and is able to cover a wider spectral range, 0.2–30 μm. More importantly, InAs/GaSb type II superlattice is based on III–V Semiconductor materials with strong Covalent Bonding. Process technologies can be applied for fast and large-scale production. This means higher yield, lower price, and better technology readiness to the market.

**Figure:**
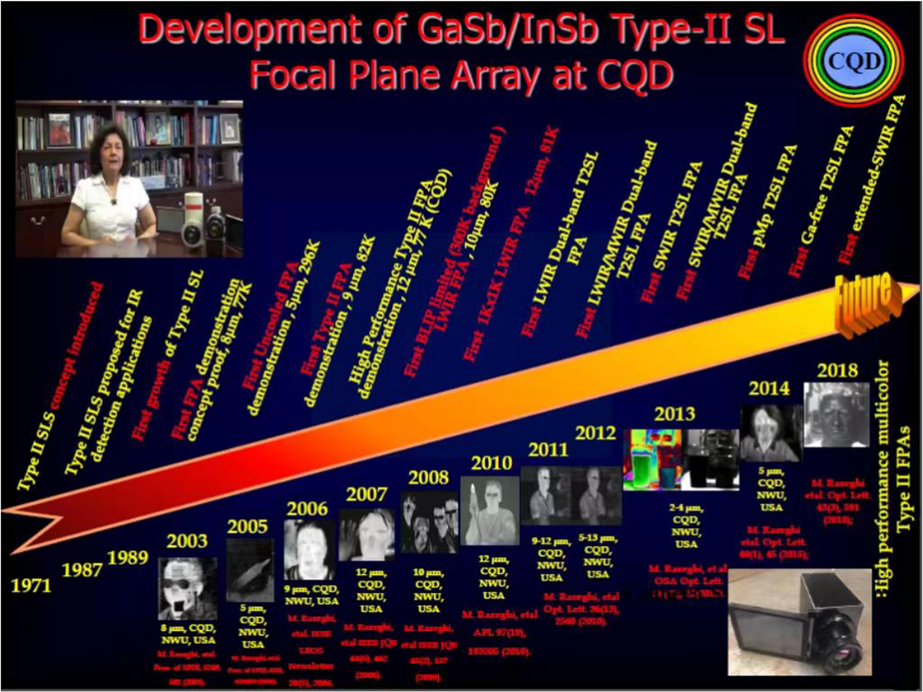
Image, from a 5-min video on YouTube, shows the timeline of development of the world’s first and best Type II superlattices for Focal plan arrays and imagers from UV to THz. She developed the device and holds the patent

**4. Your Type II Superlattice Detectors have been used on the International Space Station. Could you give us a few more examples of infrared sensor applications?**

Prof. Razeghi: Infrared sensors are now part of our everyday life. For example, Medical Thermography is the use of an infrared camera to “see” and “measure” what we call metabolism, thermoregulation or thermal energy emitted from the body. Thermography reveals a fascinating and reliable pattern of thermal activity that discloses a silent warning. These patterns can see the root cause of such conditions as headaches, allergies, dental pathologies, carotid artery disease, breast cancer, digestive dysfunction, liver/gallbladder disease, musculoskeletal conditions, pain, and immune dysfunction. An ultra-sensitive infrared video camera can even detect a gentle but visible pulsation created by blood pumping through blood vessels. This increases the demand from infrared detectors to provide higher sensitivity and speed to be able to capture small phenomena very fast.

High-performance large-format imaging sensors are highly in demand for the next generation of space, medical, persistent surveillance, and many more applications as mentioned above. Antimonide-based gap-engineered type II superlattices (T2SLs) represent the most promising material system capable of delivering a more manufacturable and affordable large-format focal plane array technology than the current technology.

Our type II superlattice detector was chosen by NASA and SpaceX for the following reasons: (1) higher operating temperature; (2) broad spectral response; (3) higher sensitivity; (4) higher stability for much less price (SWAP). Our type II superlattice detectors and imagers have been successfully applied to various space missions including NASA Landsat 9 mission and SpaceX mission just recently.

**5. You and your team have been developing uncooled infrared quantum cascade lasers in recent years. What are the advantages of such devices? What bottlenecks have you encountered so far?**

Prof. Razeghi: Recent rapid progress in information and communication technologies has exceeded our expectations for meeting the requirements of human society for different applications, such as telemedicine, in the 21^st^ century. Free-space optical (FSO) communication is one of the key technologies for realizing ultra-high-speed multi-gigabit-per-second (multi-Gb/s) large-capacity communications. Using lasers as signal carriers, FSO laser communications (Laser-Com) can provide a line-of-sight, wireless, high-bandwidth communication link between remote sites. Rapidly growing use of the Internet and multimedia services has created congestion in the telecommunications networks and placed many new requirements on carriers. IR Laser transmitters offer an intermediate low risk means to introduce desired network functionalities with extremely high bandwidth. The wireless aspect of FSO Laser-Com can be a crucial advantage, particularly in local area networks (LANs) and metropolitan area networks (MANs) where in cities the laying of optical fibers is expensive. FSO Laser-Com offers substantial advantages over conventional RF wireless communications technology, including higher data rates, low probability of intercept, low power requirements, and much smaller packaging. FSO Laser-Com systems have proven to be a viable alternative to optical fiber-based systems in several applications, as the technology comes closer and closer to providing the 5-nines (99.999%) service that many different types of users require of their data networks.

The current state-of-the-art technology in FSO communications is based around near-infrared (NIR) sources and photodetectors. Such sources and detectors are readily available at low costs due to the use of near infrared for commercial optical fiber communications. Within the NIR spectral range a major concern is the eye safety of the lasers. Although very high-power NIR diode lasers are readily available, this portion of the near-infrared wavelength range poses an extreme eye hazard due to the cornea’s ability to transmit at these wavelengths and focus infrared radiation on the eye, causing damage to the retina. Atmospheric attenuation is another major issue in the NIR FSO communication.

Quantum cascade laser (QCL) could be the best solution. QCL is a unipolar semiconductor laser based on electron-intersubband transition in a cascade fashion inside the conduction band. The emitting wavelength is determined by the subband energy spacing rather than the bandgap of the materials. This feature enables QCLs to cover a wide spectral range, from mid-IR (λ ~ 3–25 μm) up to THz (λ ~ 60–300 μm). The recent breakthrough in output power, wall-plug efficiency, and spectral tuning range is making QCL the most promising laser source in mid-IR and THz ranges.

QCL is a fast-developing technology, historical bottlenecks that were deemed to be insurmountable to overcome, like high power continuous wave room temperature operation for QCLs, were rapidly dismantled once my lab, the CQD at Northwestern University, joined the race. Actually, we are the only group in the world that can deliver over 5 W from a single QCL chip at room temperature continuous wave operation. The one remaining bottleneck, however, is that QCL in the THz range still needs cryogenic cooling due to the rather small intersubband energy spacing. Luckily, the QCL in the mid-IR range has a huge nonlinearity and can difference THz light at different frequency within the cavity at room temperature. Once we address the low output efficiency issues, QCL THz Sources will be the most promising light source in THz range as well.

**6. You have been working on the development of new manufacturing technologies, such as metalorganic chemical vapor deposition (MOCVD) outer edge technology, vapor phase epitaxy technology (VPE), Molecular-beam epitaxy (MBE), and Electron Beam Lithography (UBL). What are your expectations for these manufacturing technologies as semiconductor processing technology continues to advance?**

Prof. Razeghi: As our tools to manipulate matter reach ever smaller length scales, we are able to join in the game of discovery in the nano-world, a game that nature has long since mastered. We can get inside light, on the scale that atoms do, and create assemblies of atoms that intercept and launch photons according to the structure we design. We can shine light of any color in beams that can travel to the moon and back.

We can create crystals of matter that allow us to see even invisible light in the infrared and ultraviolet spectrum, and we can enhance our own natural senses. We can probe the human body to find cures and treat diseases. We can communicate with each other faster, over ever larger distances, sharing ever more information.

The science of Semiconductors is central to all modern quantum device physics, including electronic chips, computers, and optoelectronics, such as semiconductor lasers and detectors. The quantum semiconductor devices are involved in all areas of human Semico, trying to improve the functionality of its body and mind.

The needs of everyone in society, from food, energy, transportation, communication, entertainment, health, medicine, to exploration of space and underwater, request reliable semiconductor quantum devices in all these domains.

My goal is to make quantum devices more efficient by mimicking nature using artificial atoms and molecules. By atomic and gap engineering using different bulk crystal, novel epitaxial approaches and photolithography processes, we can create new material architectures for artificial molecules or quantum wire, artificial atom or quantum dots.

Information technology can be revolutionized by Artificial Atoms and Molecules. Artificial Active materials can produce novel Nano Sensor, nano machine, and Smart materials (with adjustable viscosity, density, elasticity...).

An example concerns the QUANTUM EYE.

Quantum-sized rods and cones containing photosensitive pigments are in the back of the eyes in retina. When light within the visible spectrum strikes these cells, nerves are fired, and the impulses are transmitted through the optic nerve to the brain with electrical signals of only 37 mev. The human eyes are capable of sensing at extremely low light levels down to a single photon. However, our vision is limited to a very narrow band of the spectrum (400–700). Our eyes have three colors sensors: red, green, blue. Humans must rely on technology to extend the limits further into the infrared and ultraviolet regions. This is why we need to make artificial eyes or Quantum Eyes.

How can we make quantum semiconductor devices? Using disruptive approaches to semiconductor epitaxy and processing. In the last decade, semiconductor technology has advanced largely in terms of electronic and photonic discrete devices, thanks to development of bulk crystal growth, and advancement in the epitaxial growth techniques such as MBE, MOCVD, Gas Source MBE. Where device quality materials can be grown with great control over composition, uniformity, and thickness, or making small devices through Photolithography.

**7. At present, technologies such as artificial intelligence and big data are profoundly changing many fields. What impacts do you think they will have on semiconductors and optoelectronic devices?**

Prof. Razeghi: Artificial intelligence and big data is built upon modern semiconductor optoelectronic technologies. They rely on Si-based logic chips to process the information, and InP or GaAs-based photonic chips to transceiver the information. They are also the driving forces for semiconductors and optoelectronic devices. The recent surge in artificial intelligence is pushing GPU to unprecedented importance while fast development in big data requires optoelectronic devices to deliver much higher efficiency and much wider bandwidth.

The Semiconductor Quantum devices are the Foundation of all modern Quantum Science, including AI, AGI, deep learning, etc.

**8. What do you imagine quantum devices will look like in the future?**

Prof. Razeghi: Our inspirations come from Nature. Fundamentally, Nature operates on the quantum level, and is full of wondrous uses of Quantum technology.

We have studied quantum wells, but Nature is not limited to a single degree of confinement. Going forward we can go more quantum…go to quantum wires, and ultimately to artificial atoms or Quantum Dots. This is not easy with current growth and processing techniques, but success here will lead to much smaller and more efficient devices. The era of 2D materials opens novel possibilities necessary to make devices smaller, lighter, and more efficient in order to revolutionize fields ranging from medicine to finance to cybersecurity.

We should also take a lesson from VLSI and silicon and go toward integration of optoelectronic components on a single wafer. By using silicon as a substrate, we can also integrate these optical components directly with the silicon electronics that drive them.

Taking advantage of the above, we need to go toward different materials with novel properties, using novel architectures with more efficient devices that operate at room temperature and that have maximal efficiency in converting energy. The era of carbon-based materials, with graphene as an example, represents infinite possibilities that are making our daily lives better.

Quantum devices will be everywhere in the future society. It will be the fundamental building block of the upcoming quantum era, as clever as Skynet (Terminator) to manipulate quantum bits for quantum computing and as delicate as BB-8 (Star Wars) to encrypt information into a single photon for quantum communication.

Since its founding in 1992, the CQD at Northwestern University has evolved from only a mere vision into a concrete world-class research laboratory, with the mission to pursue academic excellence and high-level research in compound Quantum semiconductor science and nanotechnology.

The scientific research involves developing an understanding of the physics of new semiconductor crystals for novel applications and realizing advanced semiconductor Quantum devices such as lasers, photodetectors, transistors, waveguides and switches. This entails a multidisciplinary combination of solid-state physics, quantum mechanics, electrical, mechanical and chemical engineering and materials science, as well as a strong collaborative effort between Academia, Industry, and National Laboratories. A strong testimony of the success of this endeavor has been the consistent support of several industrial corporations and government agencies from the Department of Defense to push forward the science and nanotechnology of compound semiconductor optoelectronic and quantum devices at the Center.

**Figure:**
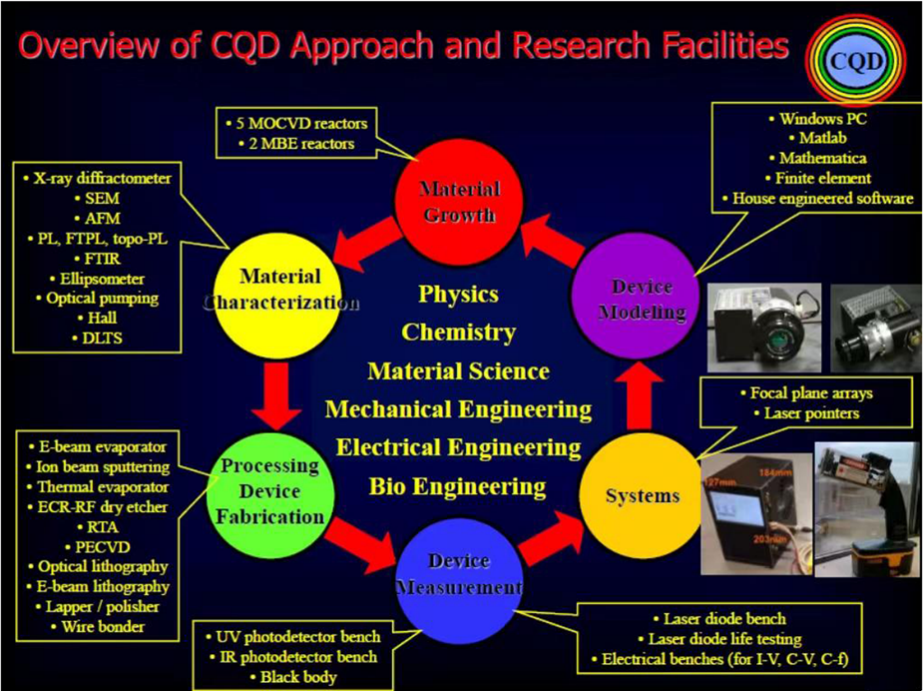
Overview of CQD approach and research facilities

**9. You went to Northwestern University in 1991 and formed the CQD there. More than 30 years later, CQD has become a world-class research laboratory, which is quite an achievement. Could you share with us the story of how you created CQD? What are your expectations and plans for the future of this laboratory?**

Prof. Razeghi: My Contribution to the world of Quantum Semiconductor is as following:

My main contribution during my career has been the development of Epitaxy on which is largely based on the field of semiconductors quantum devices.

My Initial work in this field began in 1981 (when I finished my Es-Science Dr degree in Physique from University of Paris-XI, France), when I went to work at LCR (Central Research Lab) at Thomson CSF (now Thales).

At that time, I did not know anything about semiconductors, but I was tasked with the development of MOCVD reactors and processes for InP–based lasers used in telecommunication. I became fascinated with LASER diode physics.

Many important accomplishments were made during this development. Demonstrating that high–performance quantum optoelectronic devices, in particular, laser diodes could meet the stringent requirements of optical fiber communications systems, and that high–quality GaInAsP/InP heterostructures could be grown by such a flexible method such as MOCVD, paving the way for this entire industry throughout the 80s. This work provided the foundation for my book, *The MOCVD Challenge*, which is now in its 2^nd^ edition.

**Figure:**
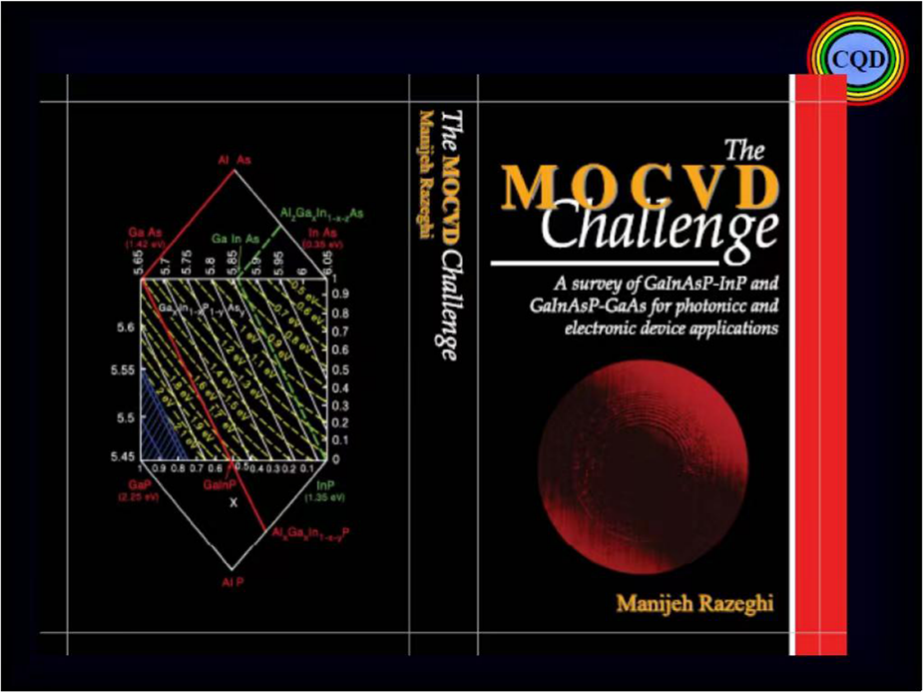
The MOCVD Challenge, 2^nd^ Edition (Taylor & Francis/CRC Press, 2010) is the combined updated version of MOCVD Challenge Vol. 1 (IOP Publishing Ltd., Bristol, U.K., 1989) and MOCVD Challenge Vol. 2 (IOP Publishing Ltd., Bristol, U.K., 1995), discussing some of Prof. Razeghi’s pioneering work

This experience allowed me to develop similar epitaxial growth equipment for metal organics and gas source molecular beam epitaxy (MOMBE, GSMBE), as well as VPE reactors. Using these techniques, I could demonstrate high-quality quantum semiconductor materials and devices.

This discovery led to my recognition as an expert in semiconductor MOCVD and in the fundamental physics and quantum devices these semiconductor materials allowed.

Although working in industry had provided me with the opportunity to discover and create new things, I missed the academic environment and wanted to use my knowledge not just for research but also to educate the next generations of scientists and engineers. After considering many offers, I chose to move to Chicago and become a Professor at Northwestern University where I was promised the opportunity to build my own unique facility to pursue groundbreaking science. This facility is the CQD.

**Figure:**
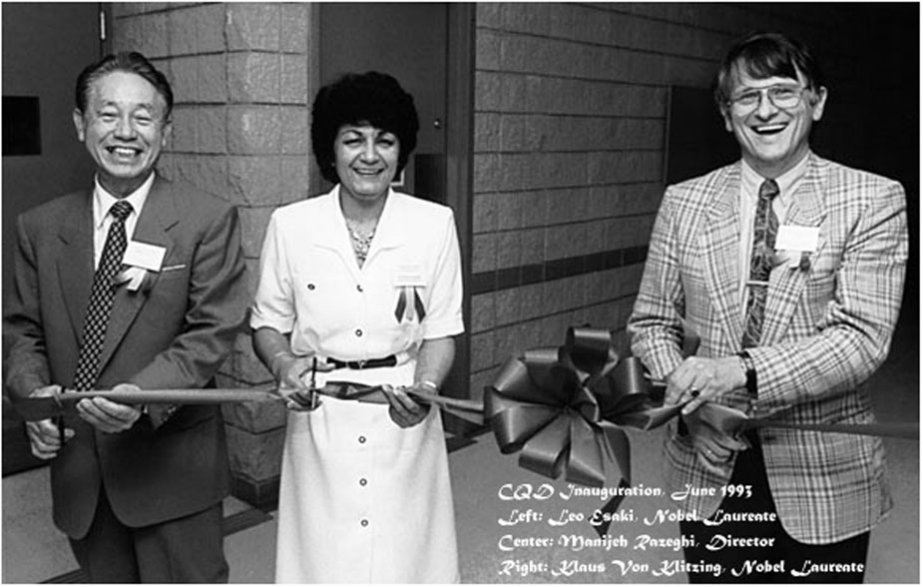
Inauguration and Ribbon cutting of CQD on June 6, 1993, with 2 nobel laureates in physics, Dr Leo Esaki (1973) and Dr Klaus Von Klitzing (1985) at Northwestern University

**Figure:**
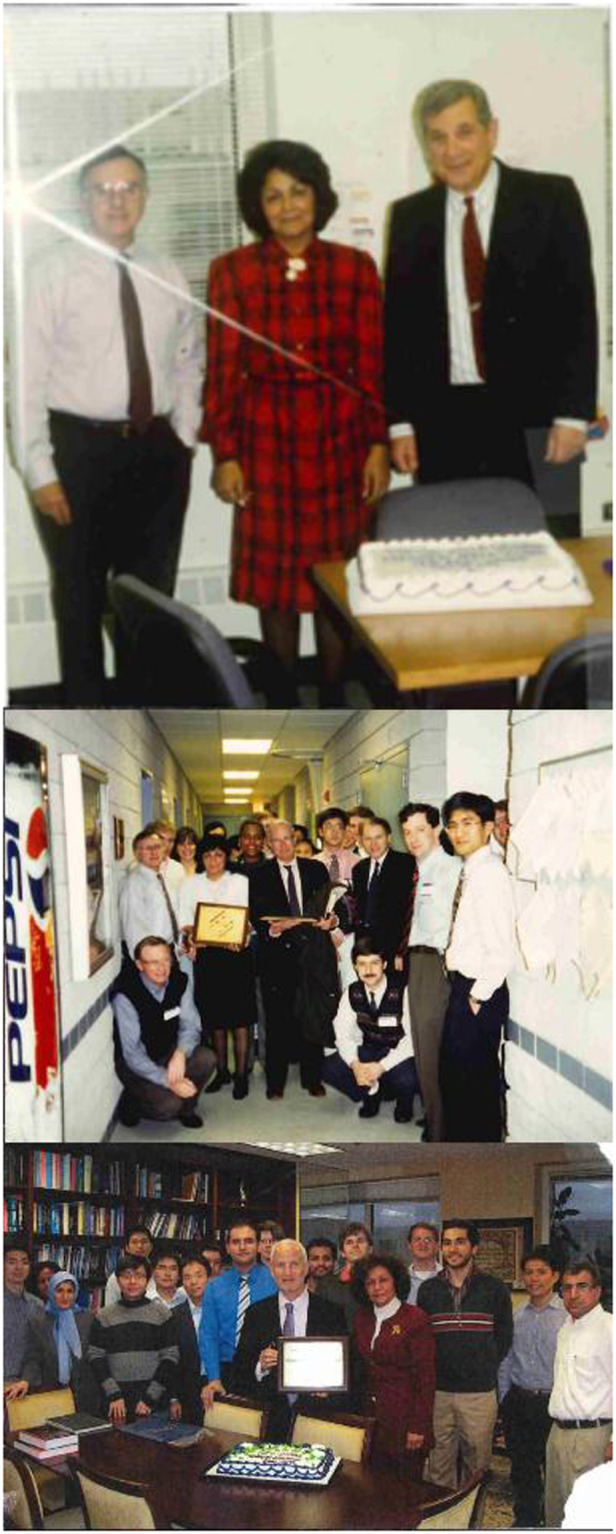
The first, second and the third president of Northwestern University visiting the CQD

My research at CQD has advanced the frontiers of Quantum semiconductor Science and nanotechnology, from creating and using forms of light in the ultraviolet, infrared, and terahertz frequency range.

I built my own unique facility to pursue groundbreaking science. CQD is a unique combination of materials and device research that enables the rapid transition from concepts to technology demonstration.

In addition to world-class facility CQD, I have also built a strong educational base by which I can transfer this knowledge to new generations of researchers.

I developed the solid-state engineering curriculum at Northwestern University as well as four textbooks, amongst which *Fundamental of Solid-State Engineering*, 4th editions (more than 2 million chapters downloaded last year). Some of the world’s first and best Semiconductor laser diodes from deep UV to THz were born at CQD. Some of the world first and best Quantum Semiconductor detectors and imagers were developed and are continuingly so at CQD, as well as technology transfer through educating great students, mentors, and leaders for the world.

Since the dawn of artificial light source 130 years ago, engineered materials have been the key to producing light. From incandescent bulbs to lasers to photonic quantum computers, the complexity of materials has accelerated to producing light with ever more control for ever more refined applications, bounded only by the creative power of the human mind.

The continuation of fundamental research at CQD and around the world will delve into the important task of advancing quantum semiconductor technology, that moves and agitates in the generation of, or responses to, the light (photons), illuminating our minds toward the next revolution of understanding ourselves and universe!!!

**Figure:**
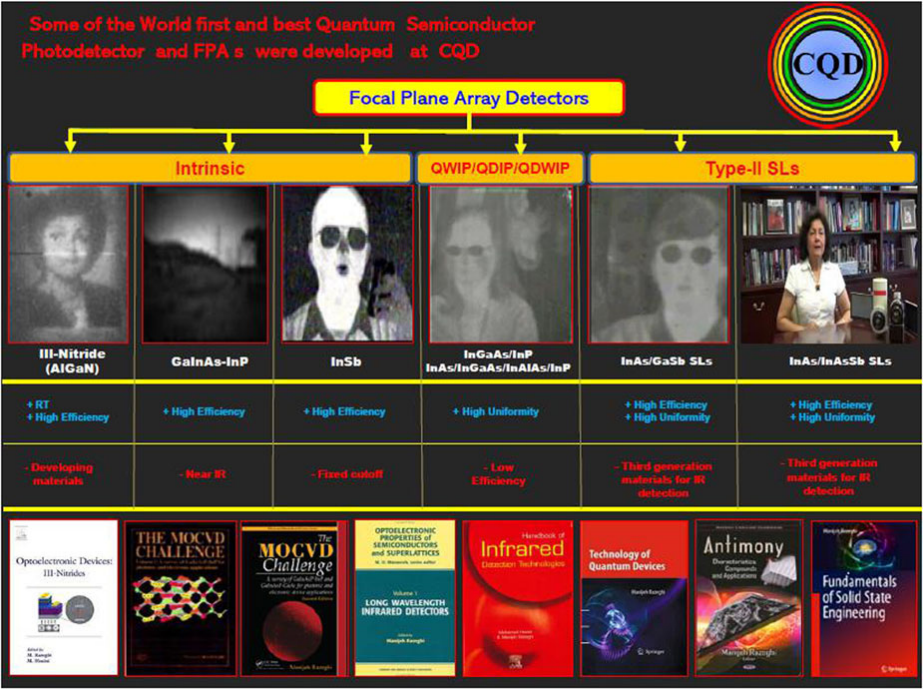
Quantum semiconductor detectors and imagers at CQD

**Figure:**
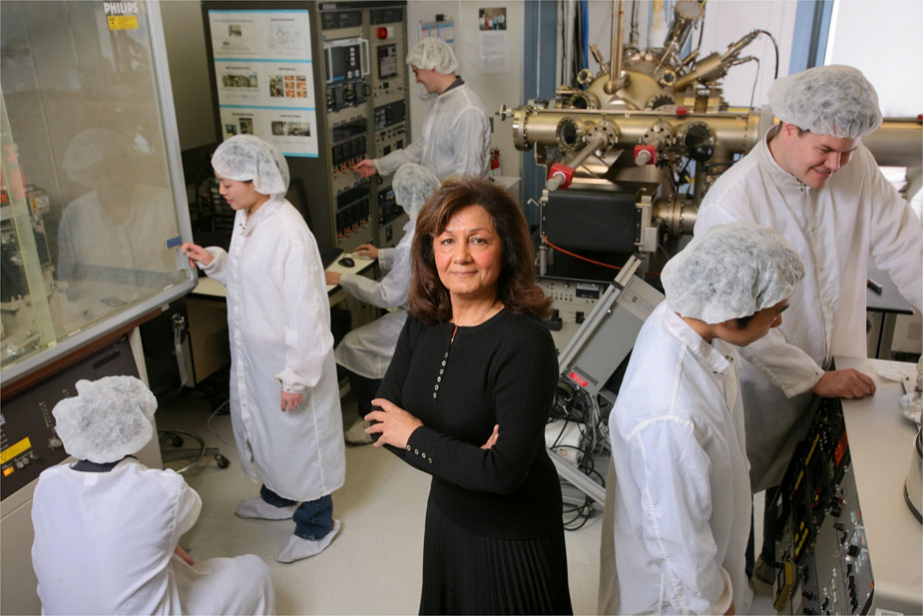
Prof. Razeghi with some of her students in Solid Source Molecular Beam Epitaxy lab

**Figure:**
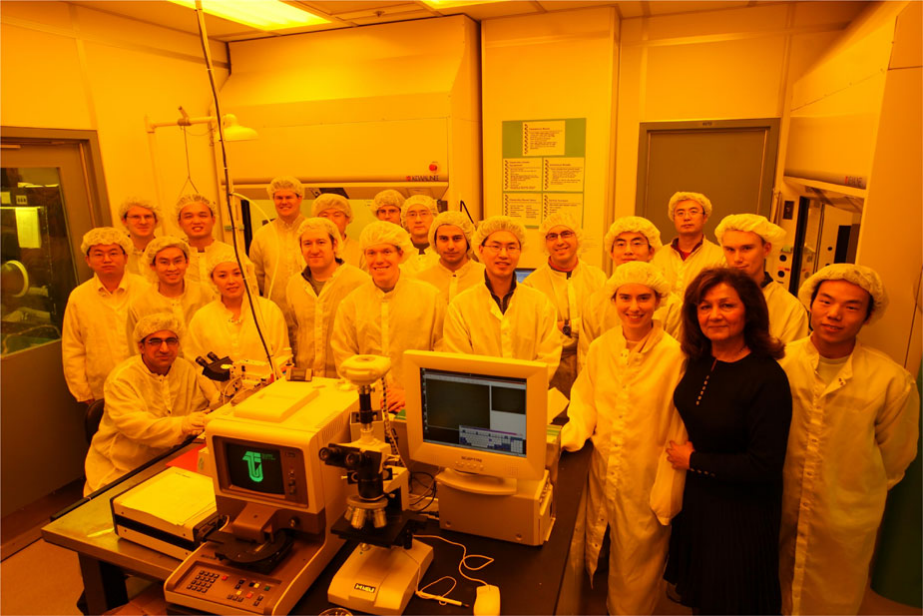
Prof. Razeghi teaching some of her students and post-Dr the processing and photolithography in a clean yellow room designed by her in 1992

**Figure:**
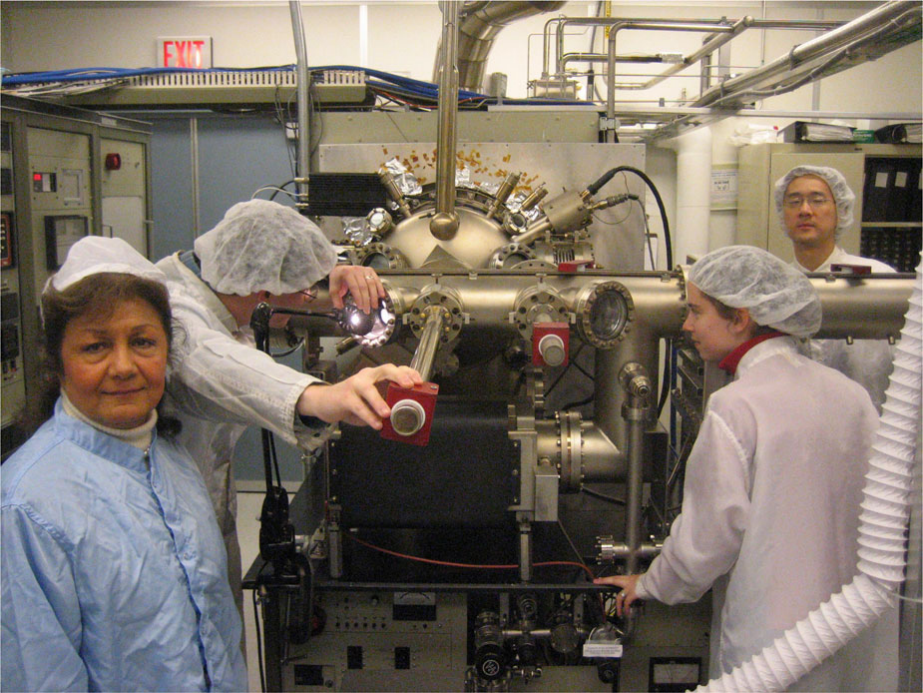
Prof. Razeghi teaching some of her students (The Gas Source Molecular Beam Epitaxy Reactor that she designed for the growth of GaInAsP/In AlAs/In P for QCL lasers and QWIP infrared detector and imagers in 1992.)

**10. When did you first have the wish to become a researcher? If you had not become a scientist, what would you have done instead?**

Prof. Razeghi: Since I was very young, I was curious about everything, such as light and rainbow: Why is a rainbow filled with such beautiful colors and in its curved shape? Later I learned that the foundation of everything, including light itself, starts with an atom. So I was, I am, and I will continue for the rest of my life, to search for the secret of life!

If I was not a scientist, I would have been devoted to education or medical practice, teaching people, or curing them (as I do now)!!!

**Figure:**
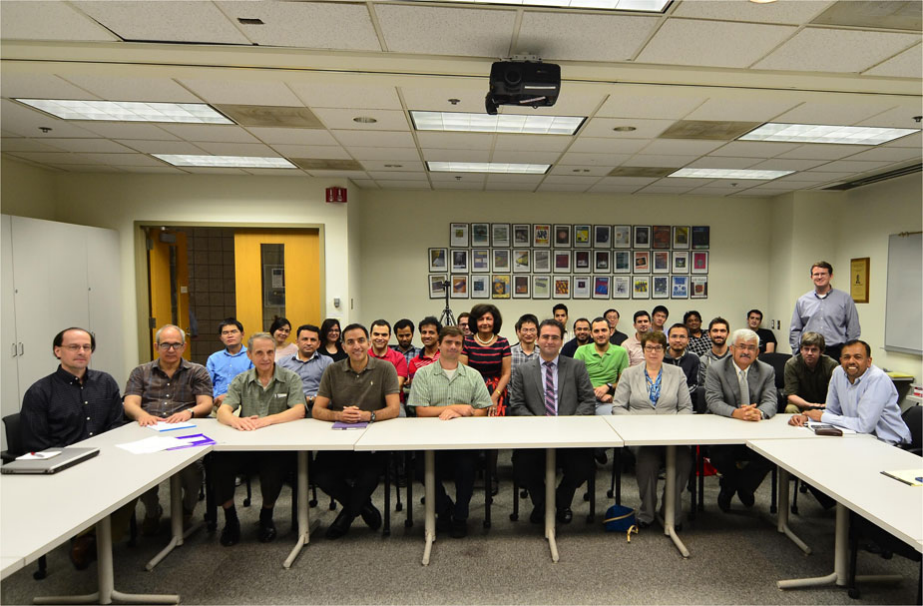
For PHD exam of her students, Prof. Razeghi inviting the experts and leading scientists from Gov Agencies, Industries and Academia

**Figure:**
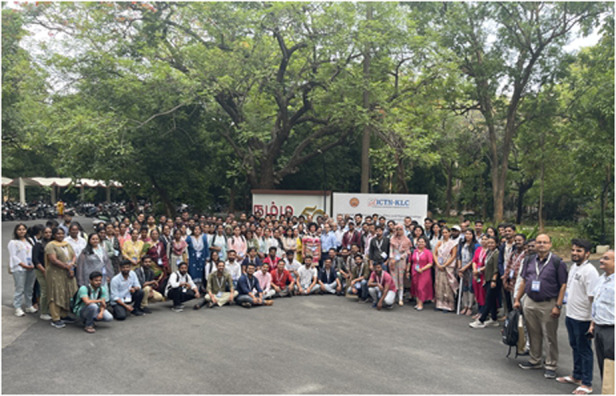
Prof. Razeghi as a plenary speaker in international conference in Nanotechnology in Indian Institute of Technology Madras in July 10–12, 2023

**Figure:**
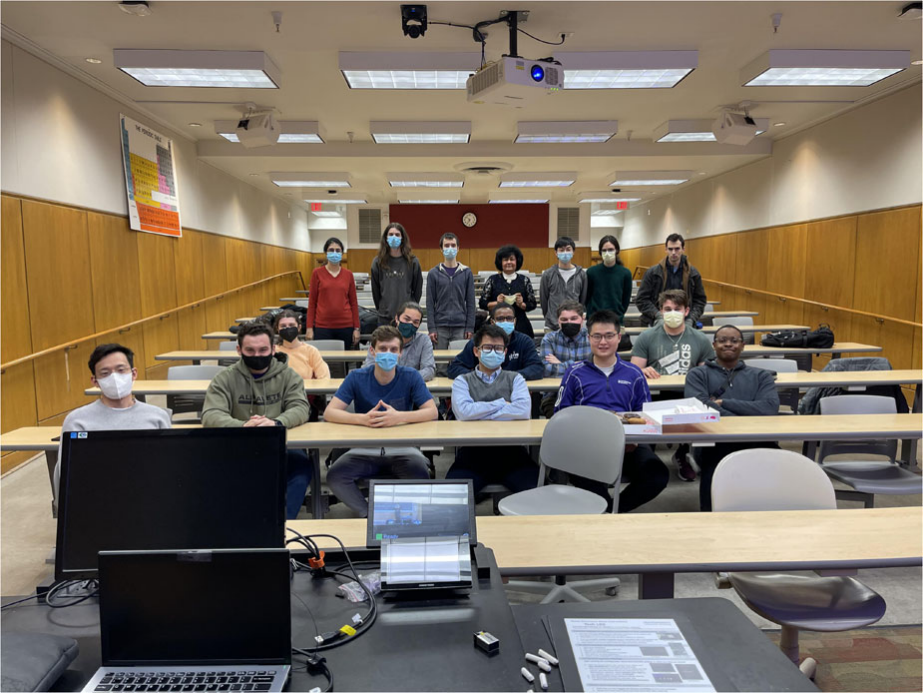
Typical Nanotechnology graduate class by Prof. Razeghi with her student during pandemic

**11. You have participated in the publication of 20 academic monographs, providing quality textbooks in the field of semiconductors and optoelectronic devices. How do you balance your work between research and academia?**

Prof. Razeghi: I would like to acknowledge my parents, my teachers, and my family, all of whom have played a vitally important role in my life and the success of my carrier. These three groups of people are perhaps the most important influences on any individual in this world.

I was only 15 years old when I met my husband, despite the protests of my parents who wanted me to finish my studies first, I got married. I managed to raise our 3 children while continuing my studies to the highest level, in other words achieving a PHD and SC. Doctorate in physics from the best university in Paris.

I would like to say I am proud to be a mother and to have raised 3 children, so that they in turn have become examples to many others. I am proud to be a professor and to be involved in the education of many excellent scientists, both female and male, who are now in many important positions in industry and academia and educating others not only in the United States, but all over the world.

**Figure:**
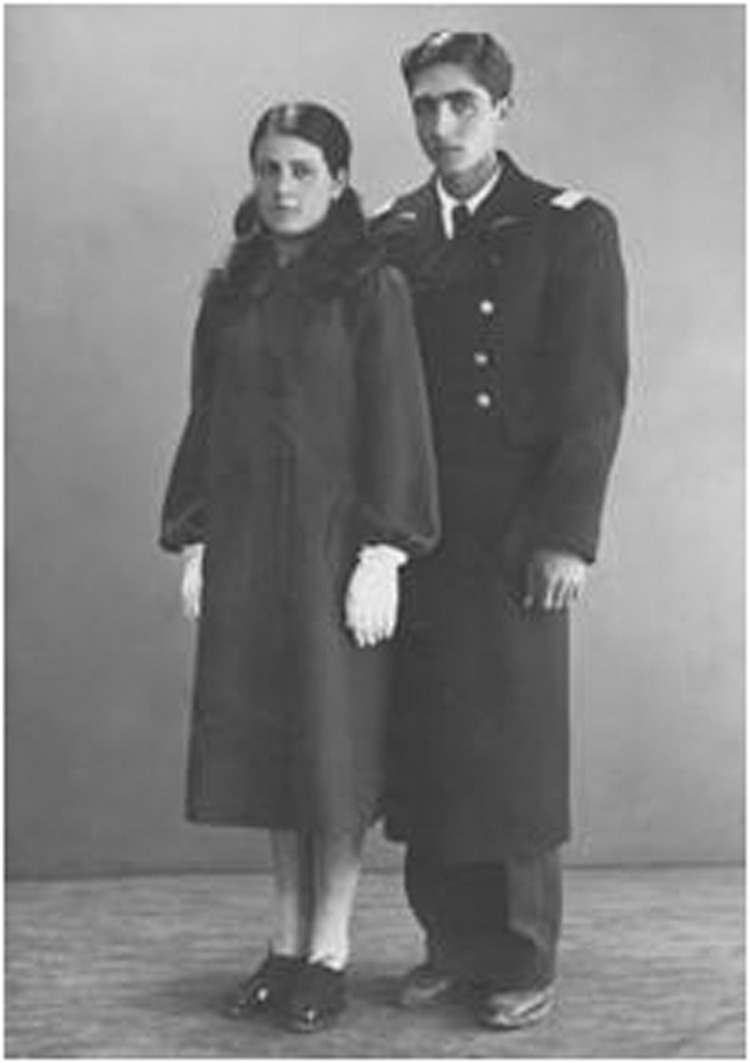
Portrait of Prof. Razeghi’s parents: her father, a 19-year-old pilot and her mother at only 14. Prof. Razeghi was their first child

**Figure:**
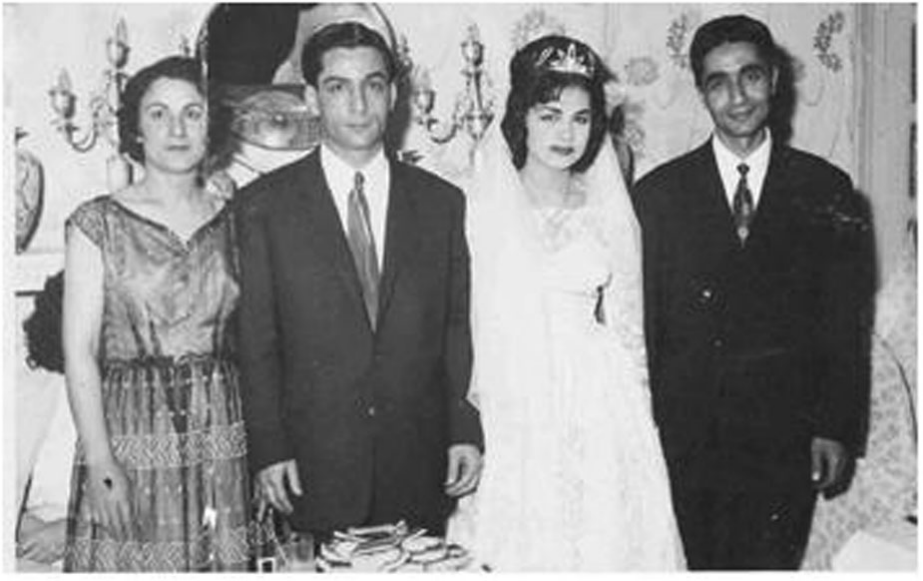
Prof. Razeghi followed her parents’ example and married at the age of 15. Here she is seen with her mother (first left), her husband (second left) and her father

**Figure:**
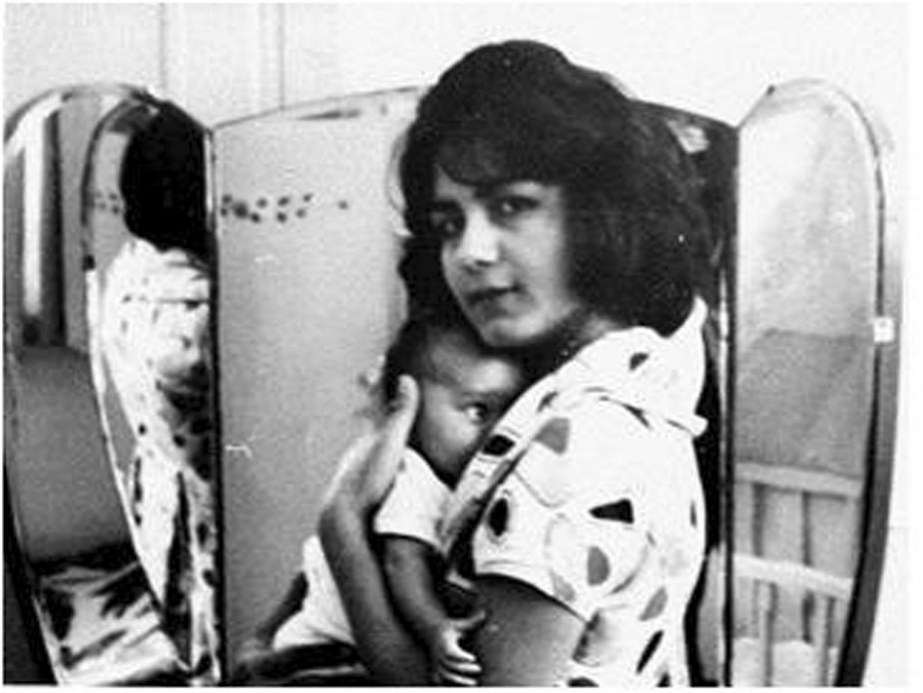
Prof. Razeghi gave birth to her first child Nader when she was 16 years old and a 10th grader

**Figure:**
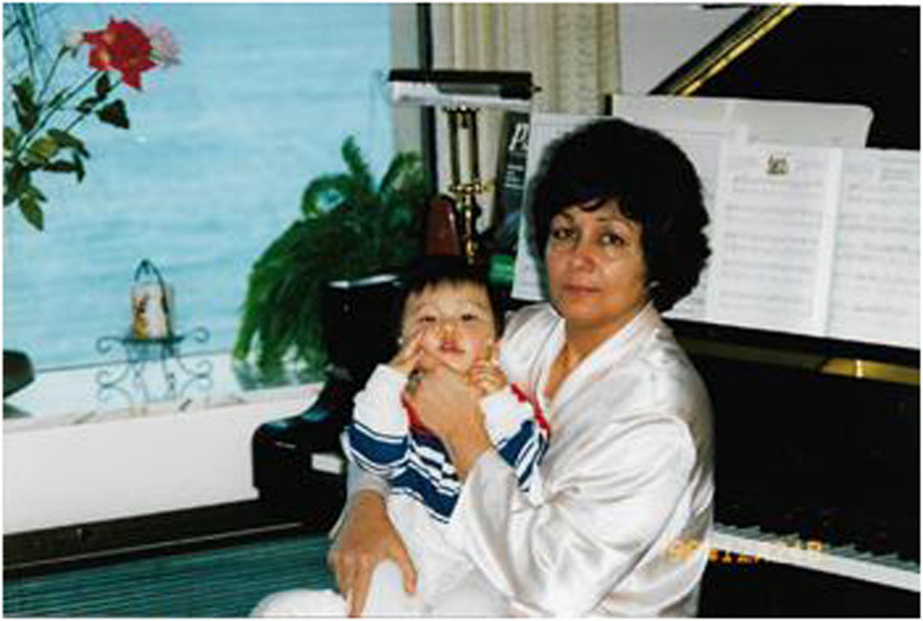
Prof. Razeghi tried to teach her first grandchild Charles to play the piano

**Figure:**
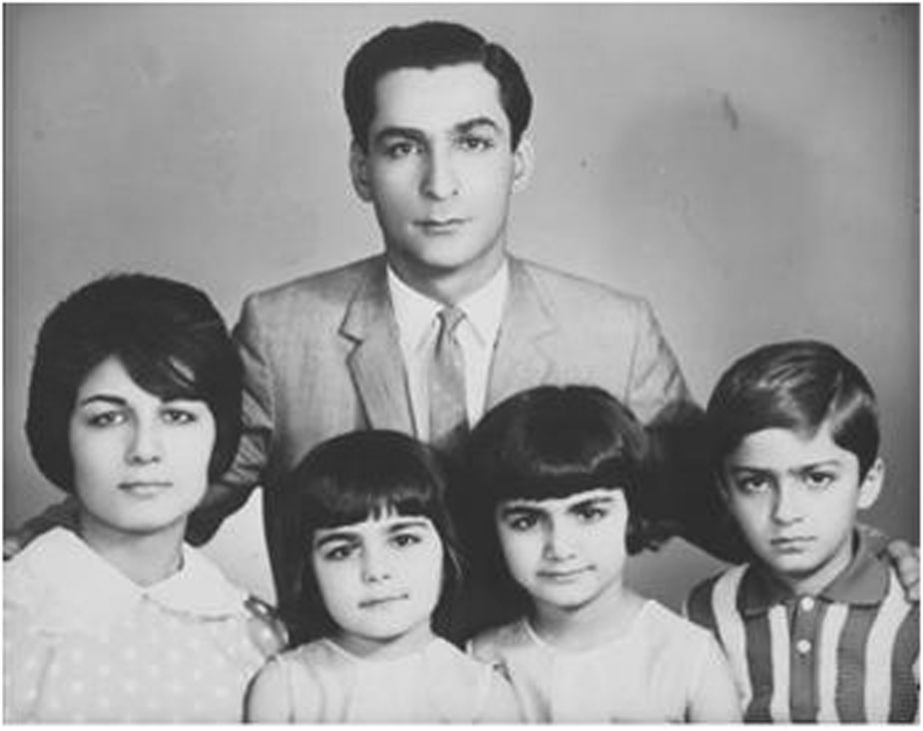
The photo of Prof. Razeghi’ s family, her husband and their 3 children Ferechteh, Farzaneh and her son Nader

**Figure:**
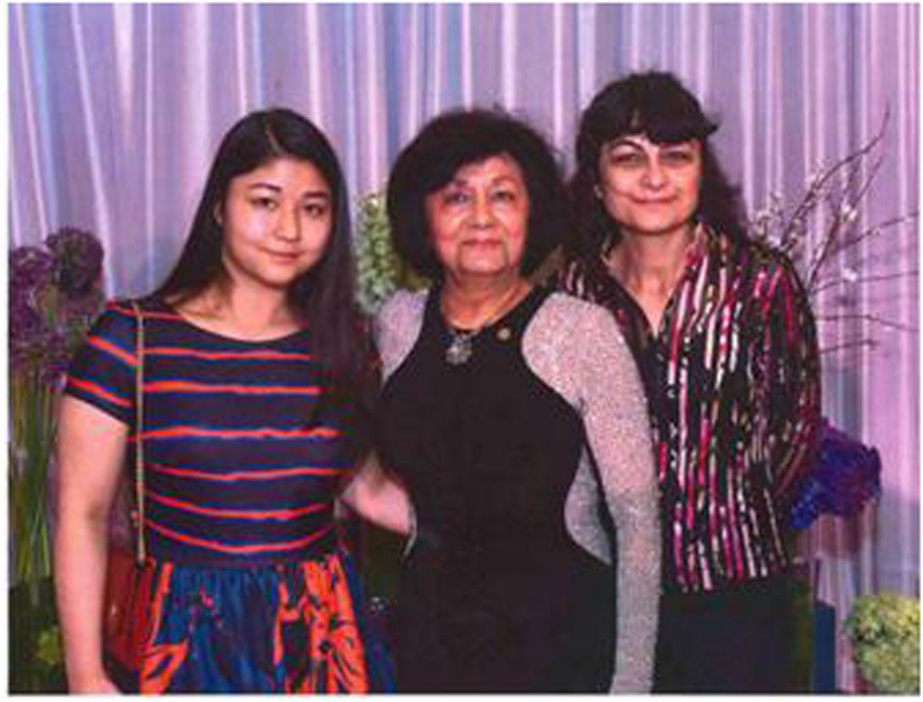
Prof. Razeghi with her daughter Ferechteh and granddaughter Julie at the Benjamin Franklin Award Ceremony in Philadephia, April 2018

I am proud to be a scientist and researcher and to be actively involved in the process of science in this world.

Lifelong passion, discipline, hardworking, and curiosity have fueled my breakthrough discoveries in Quantum semiconductor science, using light to create world changing technologies, as well all of my publications and books related to my research.

There were times when no one believed I could create such advanced devices, books etc., but with hard work and a passion for learning and exploring new ideas I have shown the impossible to be possible.

At this time, I would like to encourage all, especially women to be aware of their values, to understand their responsibility and their importance for the present and future of this world!

Research and academia are like Yin and Yang too, their interaction will maintain the harmony of my research and to influence everything within it.

I prioritize what I do. First my duty (academia), and then second my hobby (research).

**12. In August this year, you attended the Light Conference Week 2023 held in Changchun and were invited to give an academic report. How do you feel about this event? Could you tell the story of your relationship with** ***Light: Science & Applications*** **(LIGHT)?**

Prof. Razeghi: Light Conference was one of the best conferences I ever attended. Every aspect is so well organized and well served. I am so happy and proud to meet there many past members of CQD (my labs) who are now renowned experts in China and worldwide.

Light Conference for me is a part of my own scientific family and my objective is to make LIGHT the most prestigious journal in the world: To become the first choice for the submission of greatest results related to Quantum Science and Technology in the WORLD!

**Figure:**
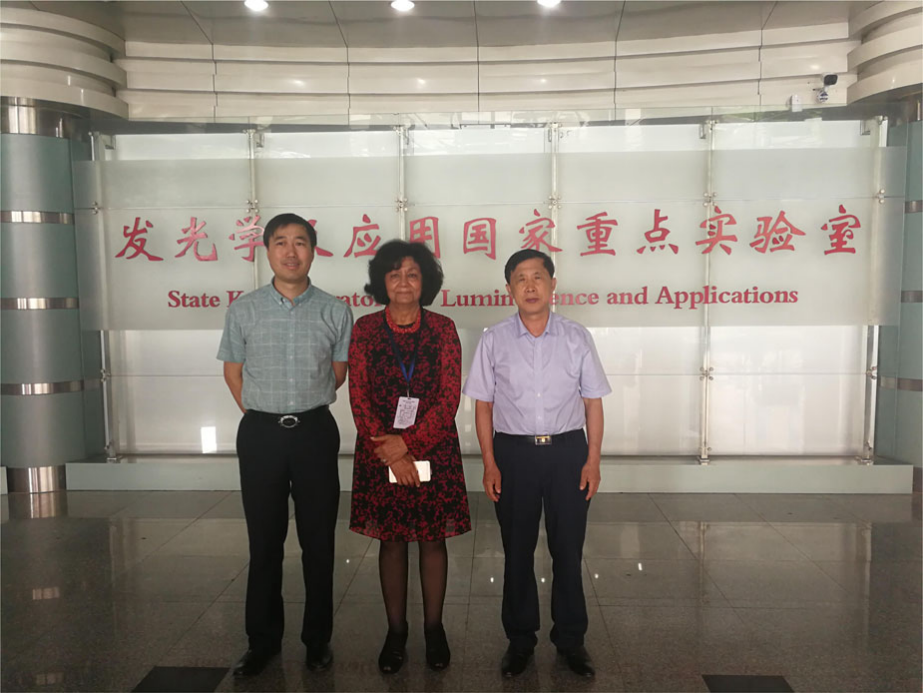
Prof. Razeghi with Academician Lijun Wang and Prof. Cunzhu Tong

**Figure:**
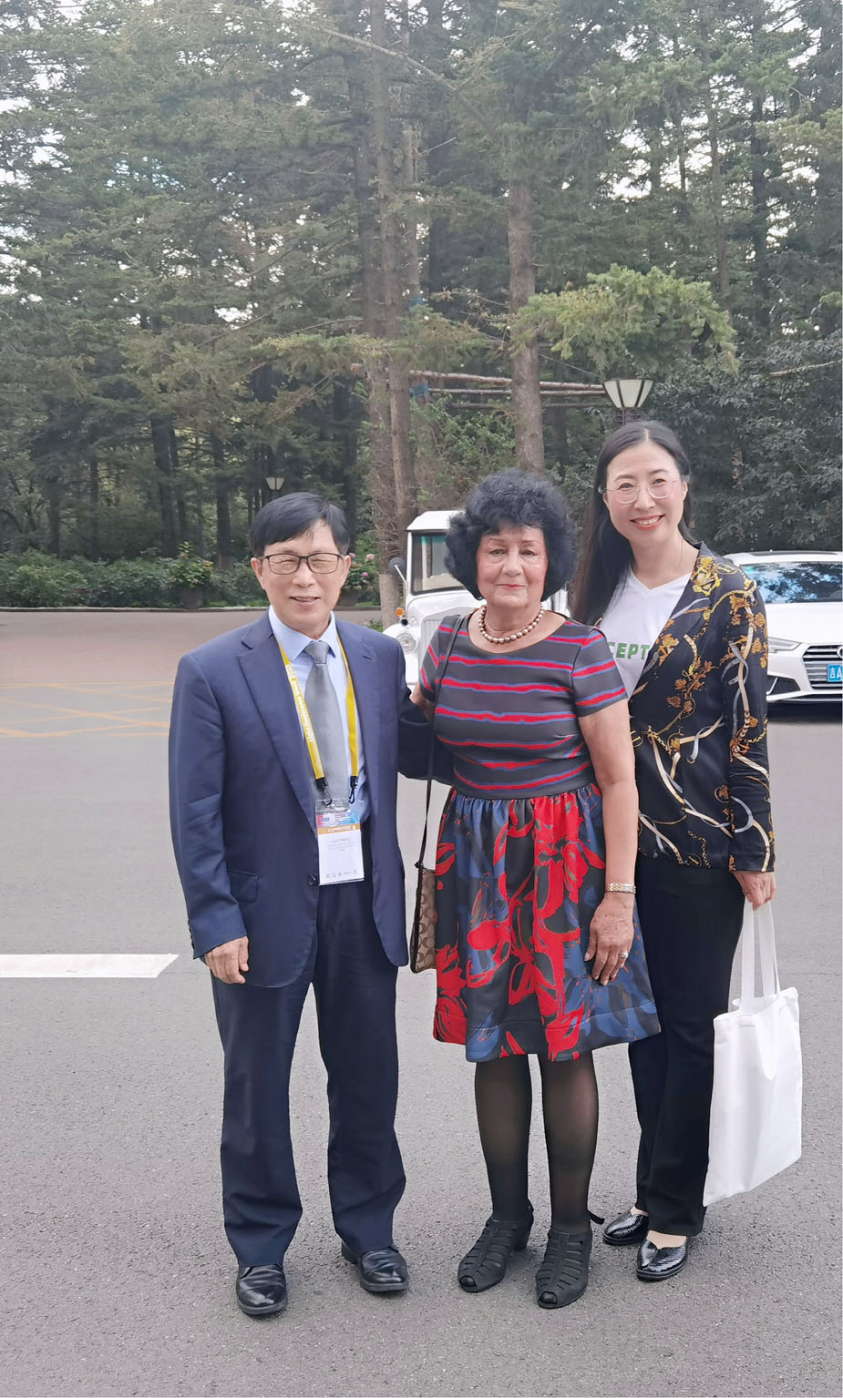
Prof. Razeghi with Academician Lijun Wang and Ms. Hui Wang during the Light Conference 2023 in Changchun

**13. Who is your science hero? Why?**

Prof. Razeghi: As a Persian (the oldest civilization in the world), my scientific hero and inspiration comes from Nature. Molana, a Persian poet, wrote “Look inside the tinniest particle you will find a universe inside”. So my hero is the creator of Nature. I try to understand and discover the secret of life by doing research to understand ATOM through Quantum mechanics, and as Niels Bohr one of the inventor of Quantum mechanics mentioned, “anyone who is not shocked by Quantum Theory has not understood it”.

I believe that once we can understand the atom of Hydrogen (smallest atom with one proton and one electron), we can understand the world.

**14. Do you have any hobbies?**

Prof. Razeghi: I like to cycle and enjoy nature while biking.

I enjoy running when the weather is good.

I play the piano, when I am struggling to solve a scientific problem.

I like to listen to lectures by different Nobel Prize Laureates related to my area of research.

I like to read scientific books.

**15. What, in your opinion, are characteristics a good science worker should have? What advice and expectations do you have for today’s young researchers?**

Prof. Razeghi: The first piece of advice I have is for parents: Education for everyone starts from the first day at home with their parents. So, we need young parents to be educated. The first building block for everyone in society starts at home. I would like to say to those of you who are parents, and to future parents, that the role of parenting and in particular education, starting from the earliest days is, was, and will continue to be crucial in all generations and cultures.

The second building block is at school, where the teachers have an important role in the education of everyone, to teach them about having discipline, honesty, curiosity, hardworking, and developing their interest and talent toward some useful art and science.

We need to have excellent teachers and knowledgeable professors to encourage the students and open their eyes to the infinite and exciting world of science.

And finally, the third building block is the family values for the success of each individual in society’s career.

In conclusion: my advice is: enjoy and like what you are doing, let your research become your hobby, try to have discipline, honesty, motivation, purpose, curiosity, working hard, to be productive. Be aware of the importance of what you are doing, and why you are doing it.

Believe on yourself and use the potential that God Gave to you.

Science and learning have no limit, the more you learn, the more there is to learn, and that is the secret of most distinguished Nobel prize winners such as Leo Esaki (Nobel prize in physics in 1973). Ninety-nine years old but still, when you listen to him, you become inspired by his vision and energy for learning, discovering the unknown world of materials science and Quantum world of Nature!!

Finally, light is the foundation of the universe.

Let there be light!

Let there be curiosity.

Let there be research.

Let there be knowledge.

